# Effects of virtual reality exposure on postural control: a comparative analysis before and after exposure in individuals with binocular dysfunction and normal vision

**DOI:** 10.7717/peerj.21169

**Published:** 2026-05-04

**Authors:** AeJin Park, HyunGoo Kang, Sang-Yeob Kim

**Affiliations:** 1 Department of Medical Health Science, Kangwon National University, Samcheok, Republic of South Korea; 2 Department of Optometry, Catholic Kwandong University, Gangneung, Republic of South Korea; 3 Department of Optometry, College of Health Science, Kangwon National University, Samcheok, Republic of South Korea

**Keywords:** Virtual reality, Postural control, Binocular dysfunction, COVD-QOL, VRSQ, Sway-path length, Sway velocity, Sway ellipse area

## Abstract

**Background:**

Non-strabismic binocular dysfunction can cause visual discomforts such as intermittent diplopia, blurriness, and asthenopia. This study investigates the impact of binocular dysfunction on cybersickness and postural control during Virtual Reality (VR) viewing, providing valuable insights for VR technology development.

**Methods:**

A sample of 46 young adults (mean age 22.3 ± 1.98 years; 22 males and 24 females) was divided into a binocular dysfunction group (23 individuals) and a normal group (23 individuals) for comparative analysis. The binocular dysfunction group consisted entirely of individuals with exophoria, indicating a convergence-related fusional deficit rather than an accommodative problem. Participants underwent visual function assessments and completed the Ocular Visual Discomfort and Quality of Life (COVD-QOL) survey, and the Virtual Reality Sickness Questionnaire (VRSQ). Postural control was evaluated using the BtrackS balance plate.

**Results:**

Results showed that the binocular dysfunction group exhibited significantly greater near exophoria, lower accommodative amplitudes, and reduced positive fusional vergence at both distance and near (*p* < 0.05). They also reported higher visual discomfort (COVD-QOL, *p* < 0.05). After VR exposure, cybersickness significantly increased in both groups (*p* < 0.001), with a greater rise in the binocular dysfunction group. This group also had higher oculomotor disorder and disorientation scores pre- and post-exposure. Postural instability significantly worsened after VR in the binocular dysfunction group (*p* < 0.05), and cybersickness severity positively correlated with in-creased medial-lateral sway and sway ellipse area. In conclusion, binocular dysfunction exacerbates cybersickness and impairs postural control, representing a critical human factor in VR environments. These findings underscore the importance of developing optical technologies to mitigate the risks associated with binocular dysfunction, thereby enhancing user safety in VR.

## Introduction

Virtual reality (VR) is a technology that stimulates human senses through computer technology to create a three-dimensional virtual reality that resembles the real world (Won, 1997). The Head Mounted Display (HMD, a wearable device placed on the head that presents separate images to each eye) system, which can create two images, one for each eye, to generate slight binocular disparity to create a realistic virtual reality environment, has become widely distributed and more user-friendly not only in the fields of education, medicine, and industry, but also in households (Fuchs, Nashashibi & Lourdeaux, 1999). Various technologies, such as head tracking using gyro sensors and acceleration sensors and high-definition 360-degree videos, are incorporated in the virtual reality environment using HMD and smartphone applications to increase the immersive experience of virtual reality. However, the cyber-sickness that occurs when viewing virtual reality videos remains a major problem that hinders the qualitative elements of virtual reality technology. The most prominent theories for the cause of cybersickness are sensory conflict theory and postural instability theory (Han & Kim, 2011). The sensory conflict theory proposes that cybersickness arises when incongruent sensory inputs particularly mismatches between visually simulated motion in VR and vestibular or proprioceptive cues related to head position and acceleration are processed by the brain. In contrast, the postural instability theory suggests that cybersickness occurs when an individual is unable to maintain stable postural control for a sustained period in unfamiliar environments, such as VR, where the usual sensorimotor strategies for balance become less effective. In addition to visual discomfort, cyber-sickness can also negatively affect the postural control that is achieved through the interaction of the visual, vestibular, and somatic systems. Visual signals are sent to the brain about the body’s position in relation to its surroundings and are processed in the brain by comparing with the information from the vestibular afferents and proprioception. Without visual inputs, the body balance depends only on vestibular inputs and proprioception. This may explain the significant increase of the total track length of center-of-pressure (COP) movement and the surface area covered by COP sway present in posturography when the visual inputs were reduced by closing one eye or both eyes in Lee’s (1996) research. Therefore, if binocular function is impaired due to strabismus or amblyopia during the growth period when visual development occurs, balance control ability will also decline (Zipori et al., 2018). Additionally, visual instability in individuals with intermittent strabismus or convergence dysfunction can act as a factor that reduces physical balance (Bronstein, 1995; Anoh-Tanon, Bremond-Gignac & Wiener-Vacher, 2002). Cho, Ro & Lee (2022) investigated the relationship between cybersickness symptoms and visual function and proved the effectiveness of a training program for visual perception to alleviate these symptoms. Howarth & Costello (1997) reported a temporary myopic shift due to increased accommodative response following VR exposure. Morse & Jiang (1999) analyzed the factors affecting the visual function of viewers after being immersed in virtual reality for 20 min and found that exophoria at near increased while accommodation and AC/A ratio decreased. Moreover, Hasebe et al. (1996) reported that 25 min of stereoscopic image viewing resulted in reduced convergence ability and a transient myopic shift in refractive status. Although studies to analyze the cybersickness and visual discomfort that arise as a result of viewing a VR video are being actively conducted, efforts to expand these studies to include the ability to exercise postural control are rarely reported. Our previous study (Jeong, 2024) identified the effect of viewing a VR video on the ability to perform postural control according to optical conditions that reflect the distance between optical centers of the lenses of the VR device and the viewer’s refractive error correction status. To sum up the study results, the ability to exercise postural control was significantly impeded under conditions in which the horizontal inter-lens optical center distance of the VR device did not match the user’s inter-pupillary distance resulting in conscious visual discomfort or under conditions that experimentally induced a hyperopic refractive state. In our previous research findings, we emphasized that adjusting the optical center of the lens of the VR device to the most comfortable distance for each individual before viewing along with full correction of vision is an important procedure to minimize the visual fatigue that sets in after viewing the VR video and compensatory action for postural readjustment. Non-strabismic binocular dysfunction can cause visual discomforts such as intermittent diplopia, blurriness, and asthenopia (Scheiman & Wick, 2008). However, it may receive less clinical attention because it does not cause sight-threatening or severe ophthalmic complications compared with other eye diseases. In natural viewing conditions, the near triad response comprising vergence, accommodation, and pupillary constriction operates in a coordinated manner to maintain clear and single binocular vision at near distances. In VR environments, however, this coordination is disrupted because the accommodative demand remains fixed at the physical distance of the display, while the vergence demand varies according to the simulated depth of the virtual scene. This mismatch, known as the vergence–accommodation conflict, is a major factor contributing to visual fatigue, binocular instability, and cybersickness (Hoffman et al., 2008). Therefore, evaluating visual parameters such as accommodative amplitude, phoria, and fusional vergence is essential for understanding why individuals with binocular dysfunction may respond differently to VR exposure compared with those with normal binocular vision.

The primary objective of this study was to investigate whether individuals with non-strabismic binocular dysfunction, despite having normal vision, experience negative impacts on postural control due to functional issues such as vergence and accommodation while engaging with VR content. In doing so, the study aims to assess whether binocular dysfunction functions as a human factor influencing both cybersickness and postural control abilities during VR video viewing, thereby providing valuable reference data for the advancement of VR technology. Furthermore, this study highlights the importance of interdisciplinary collaboration between balance evaluation and optometry to enhance user safety and comfort in emerging VR technologies.

## Materials & Methods

### Subjects

Participants were recruited as general volunteers through advertisements posted on university bulletin boards and social media platforms. After enrollment, each participant underwent a standardized binocular vision examination, and they were assigned to either the normal vision group or the binocular dysfunction group based on the clinical findings. All assessments were conducted at the Vision Examination Center of the Department of Optometry, Kangwon National University. A total of 46 young adults (22 males and 24 females) participated in this study, with an average age of 22.3 ± 1.98 years. Each participant demonstrated a monocular best-corrected decimal visual acuity of 0.9 (0.043 in logMAR visual acuity) or better and did not exhibit strabismus or any ophthalmic disorders. Additionally, they were free from neuromuscular, neurological, musculoskeletal, or systemic conditions related to the body balance, and none had a history of using medications associated with these issues. Group classification was based on a combination of binocular vision assessment and symptom severity measured by the COVD Quality of Life (COVD-QOL) questionnaire. Participants scoring ≥ 20 on the COVD-QOL, along with at least one borderline binocular vision finding, were categorized into the non-strabismic binocular dysfunction group (*n* = 23), whereas those scoring ≤ 19 and without binocular vision–related symptoms were classified as the normal group (*n* = 23). Binocular vision evaluation included heterophoria, fusional vergence ranges, near point of convergence, and accommodative amplitude. However, because Morgan’s (1944) normative values represent idealized reference ranges that even asymptomatic individuals do not always meet, clinical findings alone were not used as strict diagnostic thresholds in this study. Furthermore, comparison of binocular vision measures between the two groups showed that individuals in the binocular dysfunction group demonstrated significantly greater near exophoria, reduced accommodative amplitude, and lower positive fusional vergence at near than those in the normal group. This pattern is consistent with typical characteristics of convergence insufficiency (CI) or CI with secondary accommodative strain, supporting the validity of the classification criteria used (Scheiman & Wick, 2008). This study was approved by the Institutional Review Board of Kangwon National University (KWNUIRB-2024-04-006-001). All participants were fully informed of the study’s purpose and procedures, and written informed consent was obtained prior to their enrollment.

### Evaluation Instruments

A smartphone (LM-G710N; LG Electronics, Seoul, South Korea) and an HMD VR device (VR BOSS Xtrek, SmartPia, China) were utilized to watch the VR video. The smartphone featured a diagonal display length of 154.7 mm and a resolution of 3,120  × 1,440 pixels, offering 564 ppi (pixels per inch). The VR device allowed horizontal adjustment of the inter-lens optical center distance (60–70 mm) to match the user’s interpupillary distance, and provided an approximate field of view of 100 degrees. To evaluate postural control abilities in each group, the BTrackS Balance Plate (Balance Tracking Systems, Inc., San Diego, CA, USA) was employed. This device measures body stability and has been validated to show less than 1% measurement error and excellent test–retest reliability (ICC ≥ 0.99) for center-of-pressure assessment (Goble et al., 2016). The Center of Pressure (COP) is defined as the point of application of the resultant vertical force exerted on the supporting surface. It represents the average location of pressure applied by the body on the surface and is widely utilized as an indicator of postural control and stability during standing or movement (Winter, 1995). This device can comprehensively determine the length, area, and movement pattern of the sway by converting the sway path length to centimeters (cm) and it also calculates the sway speed (Richmond et al., 2018).

### Evaluation parameters for binocular functions

Objective refraction was performed without cycloplegia using a retinoscope (Elite retinoscope Welch Allyn, Skaneateles Falls, NY, USA). Subsequently, subjective refraction was conducted at a distance of 6 m with a manual phoropter (Essilor MPH-150E; Essilor instrument, Charenton-le-Pont, France) and a 24-inch LCD eye chart monitor. After completing the cylindrical refinement, the full refractive correction was determined using the maximum plus to maximum visual acuity (MPMVA) method (Kim et al., 2015). In this method, plus power is gradually increased (or minus power is reduced) until a slight degradation in visual acuity occurs, and the most plus (least minus) spherical lens power that still provides the best visual acuity is selected as the final correction. A unilateral cover test was first performed to identify any manifest strabismus, and no participant showed a constant or intermittent tropia. Phoria test was carried out by performing a cover test and recording the point at which the subject’s phoria was neutralized to orthophoria through the alternating cover test using a prism bar. All participants demonstrated phoria responses without any manifest deviation at both distance and near fixation, confirming that all subjects were non-strabismic. Although both the alternating cover test and the Thorington test were performed during the initial examination, only the cover test values were used for analysis because the cover test is an objective assessment that provides higher reliability than the subjective Thorington procedure. For the fusional vergence test, positive fusional vergence (PFV) and negative fusional vergence (NFV) tests were conducted at longer (5 m) and shorter (40 cm) distances, and the break point and recovery point were measured, respectively. Amplitude of accommodative (AA) is measured using the push-up method. The subjects were asked to clearly observe a target at close distance by wearing fully corrected glasses, whereupon the distance to the point where the slowly moving target began to blur was measured and converted to diopters (Kim, Lee & Oh, 2012). In the near point of convergence test (NPC test), the break point and recovery point were repeatedly measured three times using a ruler by moving the target forward to the point where it appeared as two after confirming that the target the subject was looking in front of their eyes was observed as a single target with both eyes.

### Evaluation parameters for postural controls

#### Sway-path length (PL)

The sway path length (PL) is calculated as the sum of 500 values sampled over 20 s at a frequency of 25 Hz. Each value represents the inverse of the square of the difference between the center of pressure (COP) coordinates, specifically COPx2 and COPx1 (the average positions of the left and right COPs relative to the center), as well as the square of the difference between COPy2 and COPy1 (the average positions of the front and back COPs relative to the center) (Goble et al., 2021). The PL quantifies all deviations from the center of pressure during body sway, measured in centimeters (cm). This measure can serve as an indicator of body sway magnitude, with a longer sway path length signifying greater body sway.

#### Range medial lateral of sway (Range-ML)

By plotting all sampled center-of-pressure (COP) coordinates on a two-dimensional trajectory graph, the medial–lateral sway range can be quantified. This parameter represents the horizontal amplitude of sway and is calculated as the distance, in centimeters, between the maximum rightward and maximum leftward COP positions recorded during the trial (Quijoux et al., 2021). A larger range medial lateral of sway (Range-ML) indicates greater lateral instability.

#### Range anterior posterior of sway (Range-AP)

The anterior–posterior sway range is derived by analyzing the COP trajectory along the sagittal axis. This parameter represents the forward–backward displacement of the COP and is defined as the distance, in centimeters, between the maximum anterior and maximum posterior COP positions obtained throughout the measurement period (Quijoux et al., 2021). Higher values indicate greater instability in the sagittal plane.

#### 95% confidence sway ellipse area (95CEA)

The elliptical area that constitutes the 95% confidence level is defined as the ellipse sway area that encloses approximately 95% of the points with reliability and is calculated by obtaining the area of an ellipse using the semi-major and semi-minor axes derived from the measured sway path lengths. The ellipse area of the body sway is the most widely used standard for comparing body balance and is expressed in square centimeters (cm^2^) (Schubert & Kirchner, 2014). An increase in the 95% confidence sway ellipse area (95CEA) indicates that the overall region of postural sway has expanded, reflecting reduced global postural stability.

#### Distance maximum of sway (Distance-MAX)

The maximum sway distance is specified as the distance from the average pressure center, which is the center of the moving trajectory, to the farthest pressure center point. The maximum sway distance is displayed in units of centimeters (cm) (Quijoux et al., 2021). A higher distance maximum of sway (Distance-MAX) value represents a larger peak deviation from the center of pressure, suggesting greater instantaneous instability during standing balance.

#### Velocity maximum of sway (Velocity-MAX)

The maximum sway speed is defined as the highest value occurring between two zero crossing points, defined as the moments when the velocity signal crosses zero. Positive maxima are represented by anterior values on the right, while negative maxima correspond to posterior values on the left. This maximum sway speed is measured in centimeters per second (cm/s). Anterior velocity maxima to the right are represented as positive (+) numbers, whereas posterior velocity maximum to the left is represented as negative (–) numbers (Hewson et al., 2010). An increase in velocity maximum of sway (Velocity-MAX) denotes faster and more abrupt sway movements, indicating difficulties in generating timely and stable postural corrective responses.

### Survey

#### College of optometrists in vision development quality of life (COVD-QOL) questionnaire

For the experiment, participants were asked to check for subjective symptoms of discomfort due to visual function by completing the College of Optometrists in Vision Development Quality of Life (COVD-QOL) questionnaire. Participants with a total score of 20 or higher were classified as having discomfort symptoms. The COVD-QOL was developed to measure the symptoms of accommodative abnormality, vergence abnormality, or ocular movement abnormality, and has been demonstrated to have good test-retest reliability (0.921) (Maples, 2000). The COVD-QOL questionnaire consists of 19 items rated on a 5-point scale, and each item was scored by 0 for “not at all,” 1 for “rarely,” 2 for “sometimes,” 3 for “frequently,” and 4 for “always” according to the severity of their symptoms. A total score of 20 or higher on the COVD-QOL is the level at which eye abnormality is suspected and referral to a specialist is required (Maples, 2000). In this study, subjects who scored 20 points and above on the COVD-QOL and did not gain normal values for the average visual function were classified as the binocular dysfunction group. To be classified into the normal visual function group, participants were required to meet two criteria; (1) Normal binocular vision parameters: Their measured values for phoria, fusional reserves, accommodative ability, and other relevant parameters had to fall within the normal range as defined by Morgan’s criteria (Barba-Gallardo et al., 2024). (2) Normal COVD-QOL scores: Participants also needed to report a normal quality of life related to vision, as assessed by the COVD-QOL questionnaire.

#### Virtual reality sickness questionnaire (VRSQ)

A modified virtual reality sickness questionnaire (VRSQ) (Kim et al., 2018) customized for virtual reality using HMD was used in this study. The VRSQ was developed by improving the Simulator Sickness Questionnaire (SSQ) that was initially designed for the assessment of cybersickness caused by 3D (Kennedy et al., 1993). The VRSQ was used to assess the degree of cybersickness before and after VR viewing. In the questionnaire, symptoms were mainly categorized into oculomotor disorders and disorientation. Four types of oculomotor disorders were considered including general discomfort, fatigue, eyestrain, and difficulty focusing. Disorientation was subdivided into five categories including headaches, fullness of head, blurred vision, dizziness with eyes closed, and vertigo. Each item was scored on a 4-point scale ranging from 0 (no symptoms) to 3 (very severe), and the total score was calculated by summing the weighted scores of items on each subscale (Table 1).

**Table 1:** Computation of the VRSQ score. The VRSQ consists of a total of nine questions, each with a scoring scale of 0–3 points. The nine questions are divided into two categories: four oculomotor disorder items and five disorientation items. To calculate the oculomotor disorder score, sum the scores of the four questions, divide by 12, and then multiply by 100. For the disorientation score, sum the scores of the five questions, divide by 15, and then multiply by 100. The total score is the average of the oculomotor disorder and disorientation scores.

	**VRSQ symptom**	**Score**
Oculomotor disorder (1)	General discomfort Fatigue Eyestrain Difficulty focusing	[(1)/12]*100
Disorientation (2)	Headache Fullness of head Blurred vision Dizziness (eyes closed) Vertigo	[(2)/15]*100
Total	(Oculomotor disorder score + Disorientation score)/2	

### Experimental procedures

Each participant initially underwent a refraction test and was provided with eyeglass frames into which corrective lenses were inserted. Based on the results of the visual function test and self-reported visual discomfort scores (COVD-QOL), the 46 participants were assigned to either the binocular dysfunction group or the normal group, with 23 individuals in each group. The first VRSQ survey was administered before the participants viewed the VR video. A first-person trail walking simulation was used as the VR video content (YouTube, 2024). This type of simulation has been widely used in previous studies to investigate the effects of virtual environments on balance and locomotion. By using this type of simulation, we aimed to induce a more naturalistic postural response compared to static visual stimuli (Frank, Gerd & Klaus, 2009). The participants spent 3 min viewing the VR video. Immediately after viewing the video, all subjects were asked to stand barefoot on the BtrackS balance plate. After aligning both heels of the subject symmetrically along the centerline of the measuring plate, participants were instructed to place both hands on their waist before the measurement. The postural balance measurements were conducted on the plate for 20 s in accordance with the device manual (Daniel, Niyati & Baweja, 2019), with a total of three trials performed. Two trials were conducted prior to VR viewing, and one trial was conducted after VR viewing. The first trial was excluded from the analysis as it served as a practice session to familiarize participants with the measurement procedure. Consequently, only the results from the second trial (pre-VR viewing condition) and the third trial (post-VR viewing condition) were included in the final analysis. Immediately after completion of the 20-second posture assessment, the second VRSQ questionnaire was administered to the respondents while they were experiencing motion sickness. To clarify, following VR viewing, only postural stability measurements on the BtrackS balance plate and the second VRSQ questionnaire were conducted. No additional visual function parameters (*e.g.*, phoria, accommodation, fusional vergence) were assessed after VR exposure, as the protocol required immediate evaluation of cybersickness and balance responses.

### Data analysis

The data was subjected to statistical analysis using SPSS statistics ver. 24 for Windows (SPSS Inc, Chicago, IL, USA) The independent samples *t*-test was used to compare the results of the visual function test, questionnaire scores, and postural control factors between the two groups. Pearson’s correlation analysis was conducted to identify trends between questionnaire scores and postural control evaluation factors. In all analyses, the difference was considered to be statistically significant at *p* < 0.05.

## Results

### Comparison of visual function parameters and self-reported visual discomfort Scores (COVD-QOL) between the binocular dysfunction group and the normal group

Table 2 compares the differences in the visual function parameters and COVD-QOL scores between the binocular dysfunction and normal groups. First, in the case of horizontal phoria at near, exophoria was significantly higher in the binocular dysfunction group compared to the normal group (*p* < 0.05) and the total amplitude of accommodation was significantly lower (*p* < 0.05). Moreover, the break point of positive fusional vergence at distance, and the break and recovery points of positive fusional vergence at near were significantly lower in the binocular dysfunction group (*p* < 0.05), but the negative fusional vergence between the two groups was insignificant. These results indicate that the binocular dysfunction group has reduced fusional and accommodative abilities, making it more difficult to maintain a continuous focus on objects as a single, clear image compared to the normal group. The visual discomfort scores for COVD-QOL were found to be noticeably higher in the binocular dysfunction group than those in the normal group (*p* < 0.05). Therefore, it can be considered that the binocular dysfunction group experiences greater visual discomfort and stress due to visual function deficits compared to the normal group.

### Comparison of cybersickness symptom scores (VRSQ) between the binocular dysfunction group and the normal group before and after viewing VR content

**Figure 1:**
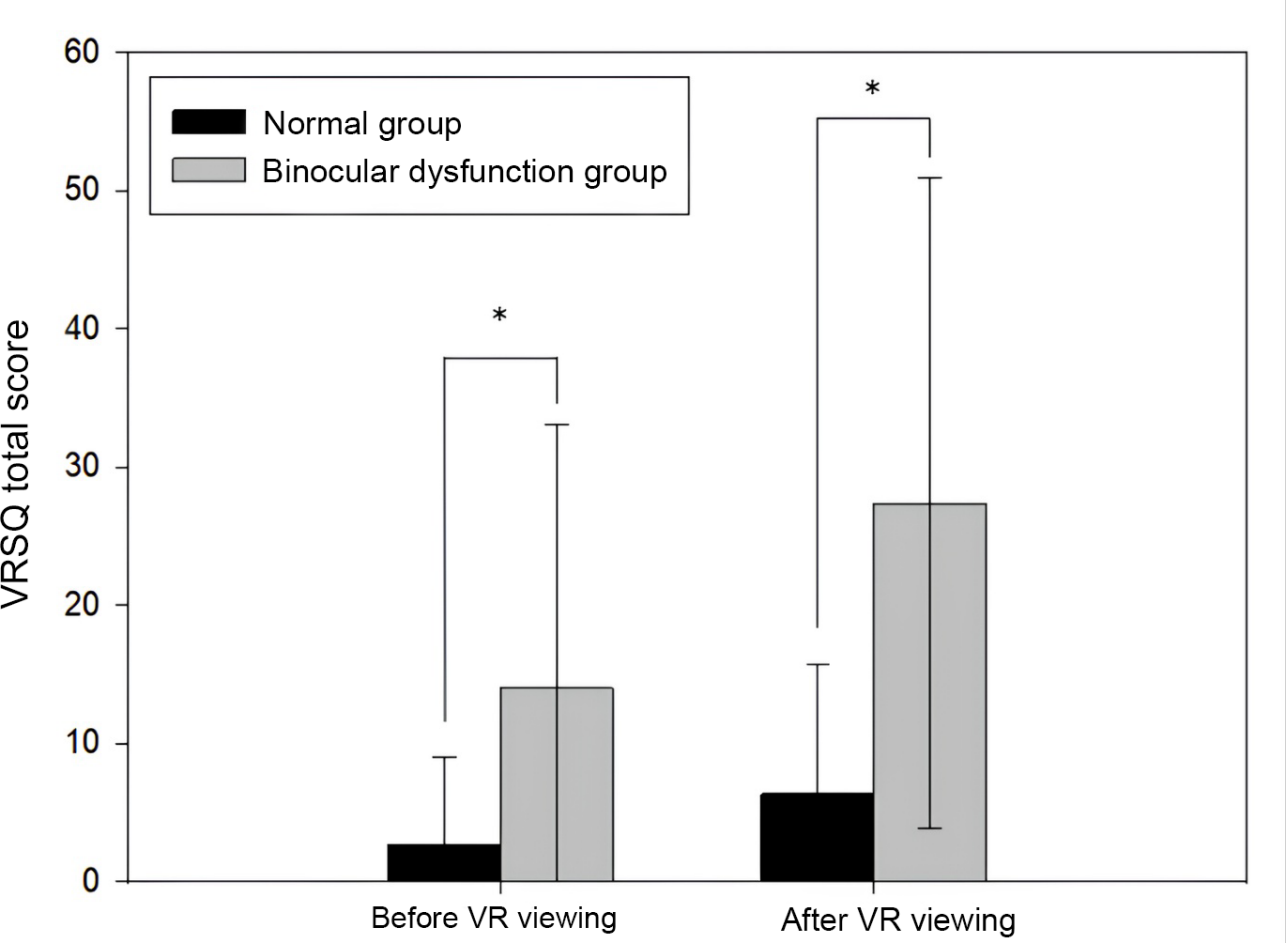
Comparison of total VRSQ scores before and after VR viewing between the binocular dysfunction group and the normal group. *p < 0.05: significantly different according to the independent samples *t*-test *N* = 23 (for each group).

The results of the comparison of the total scores for cybersickness symptoms before and after viewing the VR video in each group are presented in Fig. 1. For both groups, these scores increased significantly immediately after viewing the VR video compared to before viewing it (each, *p* < 0.001), and the increment was greater for the binocular dysfunction group. Table 3 presents the results of the more detailed analysis of the cybersickness symptoms by categorizing them into oculomotor disorder and disorientation. In the within-group analyses, neither group showed a significant change in oculomotor disorder after VR viewing (normal group: *p* = 0.367; binocular dysfunction group: *p* = 0.183). In contrast, disorientation scores significantly increased after VR viewing in both groups (normal group: *p* = 0.039; binocular dysfunction group: *p* = 0.004). Between-group comparisons further indicated that the binocular dysfunction group consistently exhibited higher levels of oculomotor disturbance and disorientation than the normal group both before (*p* = 0.016 and *p* = 0.017, respectively) and after VR viewing (*p* = 0.002 and *p* < 0.001, respectively). These findings suggest that VR content, which rapidly alters visual and spatial information, imposes a greater processing load on individuals with binocular dysfunction, making directional information more difficult to interpret compared with those with normal binocular function. In addition to the group-level differences presented in Table 2, we examined the proportion of participants who showed abnormal values relative to Morgan’s (1944) norms. In the normal group (*n* = 23), 13.0% demonstrated excessive near exophoria, 4.3% showed reduced accommodative amplitude, 21.7% exhibited reduced near positive fusional vergence, and 4.3% showed an abnormal near point of convergence. In contrast, the binocular dysfunction group (*n* = 23) showed markedly higher rates, with 60.9% exhibiting excessive near exophoria, 34.8% showing reduced accommodative amplitude, 60.9% demonstrating reduced near positive fusional vergence, and 21.7% presenting with an abnormal near point of convergence.

**Table 2:** Comparative analysis of visual function parameters and COVD-QOL scores between the binocular dysfunction group and the normal group.

**Parameters (unit)**		**Groups**		**t**	***p***
		**Normal group**	**B.D group**		
Horizontal phoria (△)	Distance	−2.00 ± 2.09	−2.70 ± 2.73	0.970	0.337
	Near	−3.96 ± 3.50	−9.15 ± 6.28	3.467	0.001^*^
Near point of convergence (D)	Break	5.28 ± 0.65	8.36 ± 8.62	−1.707	0.102
	Recover	5.78 ± 2.30	8.95 ± 9.16	−1.609	0.120
Amplitude of accommodation (D)	–	13.4 ± 2.64	11.32 ± 3.43	2.303	0.026^*^
Distance negative fusional vergence (△)	Break	9.30 ± 3.32	10.09 ± 3.49	−0.779	0.440
	Recover	5.26 ± 2.53	6.26 ± 3.37	−1.138	0.261
Distance positive fusional vergence (△)	Break	15.43 ± 4.38	13.09 ± 7.55	1.29	0.205
	Recover	8.83 ± 4.27	5.74 ± 5.27	2.183	0.034^*^
Near negative fusional vergence (△)	Break	18.52 ± 5.95	21.26 ± 9.26	−1.194	0.240
	Recover	11.78 ± 5.58	14.65 ± 8.07	−1.404	0.167
Near positive fusional vergence (△)	Break	19.57 ± 5.78	14.13 ± 9.27	2.386	0.022^*^
	Recover	11.78 ± 5.89	5.83 ± 6.83	3.168	0.003^*^
COVD-QOL (score)	–	8.09 ± 6.10	23.78 ± 13.06	−5.223	0.000^*^

**Notes.**

The (-) sign indicates exophoria, while the (+) sign indicates esophoria. B.D denotes the binocular dysfunction group.

* *p* < 0.05: significantly different according to the independent samples t-test.

*N* = 23 (for each group).

**Table 3:** Comparison of oculomotor disorder, disorientation, and total scores of the VRSQ scores before and after VR viewing between the binocular dysfunction group and the normal group.

Classification	Groups	Before VR viewing Mean ± SD (Min ∼Max)	After VR viewing Mean ± SD (Min ∼Max)	t	*p*
Oculomotor disorder	Normal	3.62 ± 9.34 (0 ∼41.67)	6.52 ± 12.04 (0 ∼50.00)	−0.912	0.367
	B.D	18.48 ± 26.11 (0 ∼91.67)	30.07 ± 31.76 (0 ∼108.33)	−1.353	0.183
t		−2.569	−3.326	–	–
*p*		0.016^*^	0.002^*^	–	–
Disorientation	Normal	1.74 ± 4.13 (0 ∼13.33)	6.09 ± 8.74 (0 ∼33.33)	−2.157	0.039^*^
	B.D	9.57 ± 14.05 (0 ∼46.67)	24.64 ± 19.12 (0 ∼66.67)	−3.047	0.004^*^
t		−2.564	−4.233	–	–
*p*		0.017^*^	0.000^*^	–	–
Total	Normal	2.68 ± 6.26 (0 ∼27.50)	6.30 ± 9.36 (0 ∼41.67)	−1.543	0.130
	B.D	14.02 ± 19.03 (0 ∼69.17)	27.36 ± 23.52 (0 ∼87.50)	−2.113	0.040^*^
t		−2.715	−3.988	–	–
*p*		0.011^*^	0.000^*^	–	–

**Notes.**

B.D denotes the binocular dysfunction group.

* *p* < 0.05: significantly different according to the independent samples t-test *N* = 23 (for each group).

Tables 4 and 5 present the results of the posture control ability of the normal group compared with those of the binocular dysfunction group before and after viewing the VR video. As is evident from Table 4, none of the postural control parameters demonstrated a significant difference between the two groups before viewing the VR video (*p* > 0.05 in all measurement parameters). However, remeasurement thereof immediately after viewing the VR video (Table 5) revealed a significant increase in all parameters measured in the binocular dysfunction group, compared to the normal group. (PL; *p* < 0.05, Range-ML; *p* < 0.05, Range-AP; *p* < 0.05, Distance-MAX; *p* < 0.05, Velocity-MAX; *p* < 0.05, 95CEA; *p* < 0.05 respectively). Importantly, the magnitude of these group differences was moderate to large, as reflected by the corresponding effect sizes (Cohen’s d: PL = 0.94, Range-ML = 0.80, Range-AP = 0.83, Distance-MAX = 0.62, Velocity-MAX = 0.61, 95CEA = 0.79), indicating that individuals with binocular dysfunction exhibited noticeably greater postural instability following VR exposure.

**Table 4:** Comparison of postural control parameters between the binocular dysfunction group and the normal group before VR viewing.

**Parameters (unit)**	**Groups**		**t**	***p***
	**Normal group**	**B.D group**		
PL (cm)	17.00 ± 3.62	18.48 ± 5.10	−1.134	0.263
Range-ML (cm)	0.67 ± 0.23	0.76 ± 0.31	−1.029	0.309
Range-AP (cm)	1.89 ± 0.60	1.80 ± 0.59	0.094	0.925
Distance-MAX (cm)	1.10 ± 0.45	1.04 ± 0.38	0.459	0.649
Velocity-MAX (cm/s)	3.38 ± 1.49	3.63 ± 1.49	−0.554	0.582
95CEA (cm^2^)	1.00 ± 0.70	1.09 ± 0.56	−0.488	0.628

**Notes.**

B.D denotes the binocular dysfunction group.

* *p* < 0.05: significantly different according to the independent samples t-test *N* = 23 (for each group).

PL Sway-path length, Range-ML Range medial lateral of sway Range-AP Range anterior posterior of sway Distance-MAX distance maximum of sway Velocity-MAX velocity maximum of sway 95CEA 95% confidence sway ellipse area

**Table 5:** Comparison of postural control parameters between the binocular dysfunction group and the normal group after VR viewing.

**Parameters (unit)**	**Groups**		**t**	***p***	**Cohen’s d**
	**Normal group**	**B.D group**			
PL (cm)	19.00 ± 5.06	26.22 ± 9.64	−3.178	0.003^*^	−0.938
Range-ML (cm)	0.84 ± 0.39	1.45 ± 1.00	−2.726	0.011^*^	−0.804
Range-AP (cm)	1.96 ± 0.66	2.80 ± 1.28	−2.802	0.008^*^	−0.825
Distance-MAX (cm)	1.24 ± 0.49	1.67 ± 0.85	−2.084	0.044^*^	−0.620
Velocity-MAX (cm/s)	3.96 ± 1.31	5.94 ± 4.44	−2.054	0.046^*^	−0.605
95CEA (cm^2^)	1.37 ± 0.91	3.20 ± 3.16	−2.670	0.013^*^	−0.787

**Notes.**

B.D denotes the binocular dysfunction group.

* *p* < 0.05: significantly different according to the independent samples t-test *N* = 23 (for each group).

PL Sway-path length, Range-ML Range medial lateral of sway Range-AP Range anterior posterior of sway Distance-MAX distance maximum of sway Velocity-MAX velocity maximum of sway 95CEA 95% confidence sway ellipse area

### Analysis of the correlation between self-reported visual discomfort, cybersickness scores, and postural control parameters

**Figure 2:**
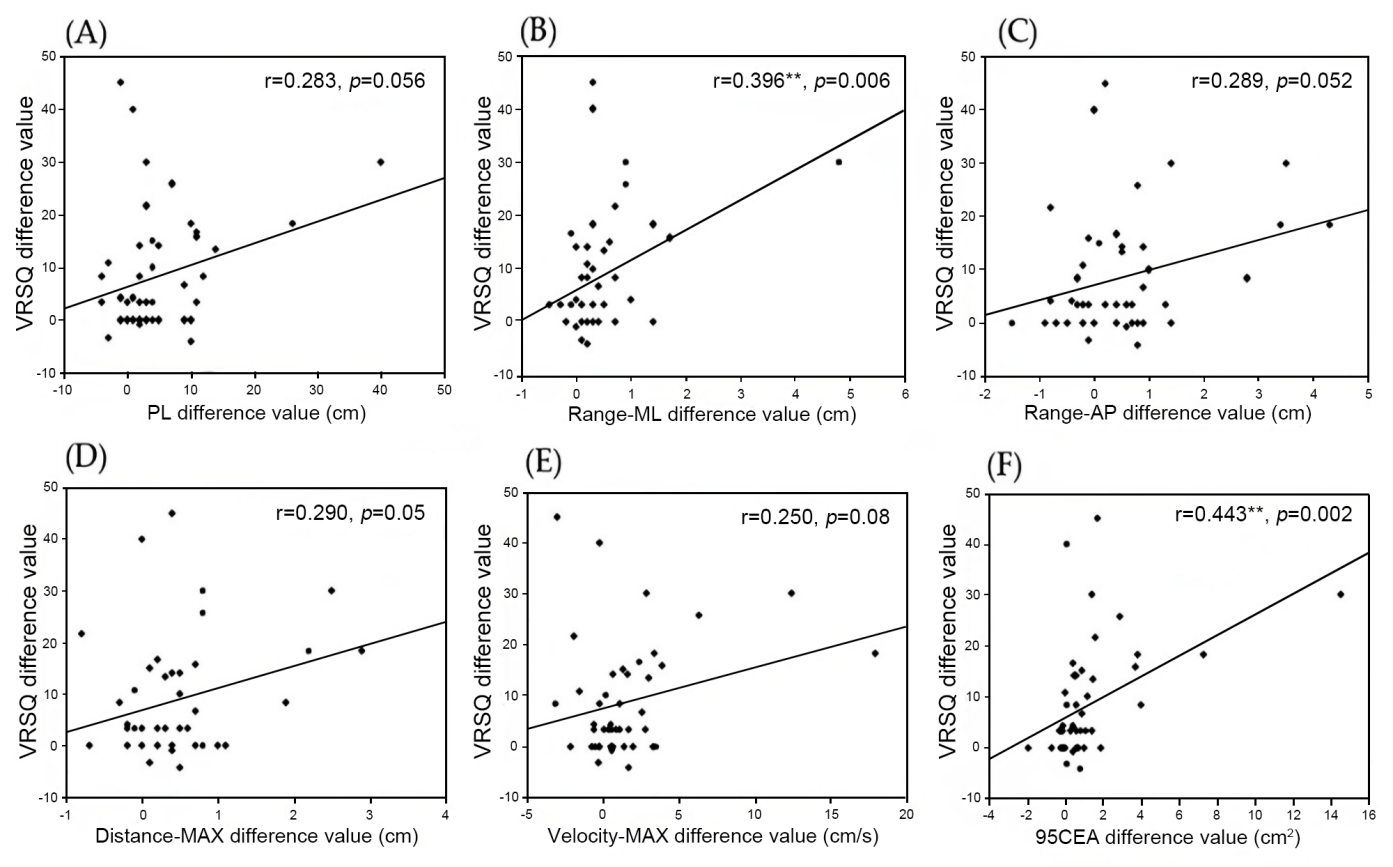
Correlation analysis between difference value in cybersickness scores and difference value in postural control parameters before and after VR viewing in all groups. *p < 0.05: significantly different to Pearson’s correlation coefficient N = 46 PL, Sway-path length; Range-ML, Range medial lateral of sway; Range-AP, Range anterior posterior of sway; Distance-MAX, distance maximum of sway; Velocity-MAX, velocity maximum of sway; 95CEA, 95%confidence sway ellipse area.

Figure 2 presents the correlations between the change in cybersickness symptoms (VRSQ difference score) and the change in postural control parameters before and after VR viewing. Significant but moderate correlations were found for the range of medial–lateral sway (VRSQ difference *vs.* Range-ML difference: *r* = 0.396, *p* = 0.006, Fig. 2B) and for the 95% confidence sway ellipse area (VRSQ difference *vs.* 95CEA difference: *r* = 0.443, *p* = 0.002, Fig. 2F), indicating that participants who reported greater increases in cybersickness tended to show corresponding increases in these specific postural instability measures. Other postural parameters (PL, Range-AP, Distance-MAX, and Velocity-MAX) showed non-significant correlations with cybersickness, indicating that VR-induced discomfort did not uniformly influence all aspects of postural control.

## Discussion

VR technology provides users with a vivid virtual environment and has found widespread application in video games, education, healthcare, *etc.* (Fuchs, Nashashibi & Lourdeaux, 1999; Han & Kim, 2011). However, relief for cybersickness symptoms such as dizziness, nausea, and vertigo after VR viewing is needed to create a human-friendly VR environment. Although technological innovation is required in various specialized areas, devising measures to minimize cybersickness is an important task in the field of optometry. Even though several studies have focused on the analysis of cybersickness and visual discomfort resulting from viewing VR videos (Howarth & Costello, 1997; Han & Kim, 2011; Cho, Ro & Lee, 2022), visual discomfort associated with the vergence–accommodation conflict has been most rigorously characterized by Hoffman et al. (2008). This prompted our study, which aimed to examine whether individuals with binocular dysfunction show different patterns of cybersickness and postural control after viewing a VR video.

A comparison of the visual function parameters and self-reported visual discomfort scores (COVD-QOL) between the binocular dysfunction group and the normal group (Table 2) indicated that the binocular dysfunction group had significantly higher exophoria at near (*p* < 0.05), and that the amplitude of accommodation (*p* < 0.05) and positive fusional vergence at near (*p* < 0.05) decreased significantly. These results are indicative of a form of binocular dysfunction with functional deterioration in the convergence and amplitude of accommodation. Typical convergence insufficiency is characterized by a larger exophoria at near, a receded near point of convergence, and reduced positive fusional vergence at near, whereas distance phoria and both distance and near negative fusional vergence generally remain within normal limits (Scheiman & Wick, 2008). However, the absence of a significant group difference in near point of convergence in the present study appears to be attributable to the substantially greater inter-individual variability observed within the binocular dysfunction group compared with the normal group. As positive fusional vergence demand increases to compensate for the larger near exophoria, reliance on accommodative convergence may also increase, which could partially counteract the extent of near point of convergence recession. This compensatory mechanism may have reduced between-group differences in near point of convergence, thereby preventing the difference from reaching statistical significance. The COVD-QOL score for subjective visual discomfort symptoms was 23.78 ± 13.06 points for the binocular dysfunction group, and this score differed significantly from the 8.09 ± 6.10 points for the normal group (*p* < 0.05). Furthermore, a comparison of the total cybersickness scores before and after VR video viewing (Fig. 1) revealed significant increases in both groups following VR exposure (*p* < 0.001). However, caution is required when interpreting the magnitude of change because the normal group exhibited pre-VR VRSQ scores close to the lower bound of the scale, suggesting a potential floor effect that may have constrained the observable change. The relatively large standard deviations observed in questionnaire-based measures likely reflect substantial inter-individual variability in susceptibility to cybersickness, as well as floor effects due to low baseline scores in some participants. Despite this limitation, it is noteworthy that the binocular dysfunction group displayed consistently higher absolute VRSQ scores than the normal group at both pre- and post-VR measurements. Since several baseline VRSQ items (*e.g.*, eyestrain, difficulty focusing) can reflect pre-existing binocular vision–related symptoms rather than VR-induced effects, the higher pre-VR scores in the binocular dysfunction group likely represent greater underlying visual discomfort associated with their functional deficits. A more detailed analysis separating cybersickness into oculomotor disorder and disorientation (Table 3) showed no significant pre–post changes in oculomotor disorder scores in either group (normal: *p* > 0.05; binocular dysfunction: *p* > 0.05). In contrast, disorientation scores increased significantly in both groups (normal: *p* < 0.05; binocular dysfunction: *p* < 0.05), and the binocular dysfunction group consistently demonstrated higher values. These findings align with previous reports that cybersickness is strongly characterized by disorientation symptoms such as headache, fullness of head, blurred vision, and dizziness (Robert, Julie & Robert, 2010). Although Regan (1995) noted that cybersickness increases linearly with prolonged VR exposure exceeding 10–20 min, the present study observed a noticeable increase in disorientation even after a brief exposure of approximately 3 min. This pattern may be partially explained by the vergence–accommodation conflict inherent to VR displays, which has been shown to induce visual discomfort within short viewing durations (Hoffman et al., 2008), particularly in individuals with reduced binocular stability. Overall, these results suggest that binocular dysfunction is associated with more pronounced visual discomfort and higher susceptibility to cybersickness, particularly disorientation. However, given the cross-sectional nature of the comparison, these patterns should be interpreted as associations rather than causal effects of binocular dysfunction on VR-induced symptoms.

Tables 4 and 5 compare the normal group and the binocular dysfunction group in terms of their ability to exercise postural control before and after viewing the VR video. First, the two groups did not differ significantly with respect to any of the posture control parameters measured before viewing the VR video (*p* > 0.05, Table 4). On the contrary, immediately after they viewed the video, significant differences were found among all the parameters (*p* < 0.05, Table 5). As the total path length increased as a result of deviating from the center of pressure, the binocular dysfunction group showed greater postural instability immediately after VR viewing compared with the normal group. Moreover, a correlation analysis was performed to examine the relationship between changes in cybersickness symptoms and changes in postural control parameters after VR viewing (Fig. 2). The results showed significant but moderate associations for the range of medial–lateral sway (Range-ML; *r* = 0.396, *p* = 0.006, Fig. 2B) and the 95% confidence sway ellipse area (95CEA; *r* = 0.443, *p* = 0.002, Fig. 2F), indicating that participants who experienced greater increases in cybersickness tended to show corresponding increases in these specific measures of postural instability. In contrast, the remaining postural parameters demonstrated weak or non-significant correlations with cybersickness, suggesting that VR-induced discomfort may selectively affect particular components of balance control rather than uniformly influencing all stability measures. Importantly, given the correlational nature of the analysis, the directionality or causality of the relationship between cybersickness and postural instability cannot be determined. This interpretation is consistent with previous work by Anoh-Tanon, Bremond-Gignac & Wiener-Vacher (2002) who reported that binocular vision problems or convergence insufficiency can contribute to reduced fixation stability and dizziness. However, although the correlations shown in Fig. 2 demonstrate meaningful relationships, the use of pre–post difference scores has inherent limitations, as this approach may increase measurement noise. In our sample, baseline VRSQ scores were extremely low, producing a floor effect that limited the interpretability of raw-score correlations. For this reason, difference scores were used as the most practical method to capture VR-induced changes, and the results should be interpreted with this limitation in mind. Alhassan et al. (2021) reported that both the positive and negative fusional vergence decreased at near after playing the VR game. The HMD of the VR device consists of a smartphone display, a (+)17 D-(+)20 D optical lens, and other components. Due to the high plus (+) optical lens within the VR headset, the user views the virtual image with reduced accommodative demand, as the focal plane is shifted to a far distance inside the VR device. In this visual environment, positive accommodative convergence corresponding to the gaze distance does not occur, and this visual condition may be related to greater reliance on positive fusional vergence (Son, Lee & Lim, 2019). To clarify, the strong plus lenses in the VR device shift the focal plane of the virtual image to a far distance, so the crystalline lens of the eye requires little accommodative effort even when the virtual scene appears to be located at a closer distance. However, the vergence system must still converge to the simulated viewing distance. This mismatch low accommodation demand but high vergence demand creates a vergence–accommodation conflict (Hoffman et al., 2008), which increases oculomotor effort and visual discomfort. This explains why greater positive fusional vergence may be required in such a VR viewing environment. In this study, the binocular dysfunction group was characterized by very low positive fusional vergence amplitudes due to the intervention of more positive fusional vergence to compensate for the high degree of exophoria. This reduced fusional vergence reserve indicates that these individuals already operate with a higher vergence burden during normal viewing. To conclude, the imbalance of accommodation and convergence caused by the internal environment of the VR device may be related to increased overall body sway by altering the vergence signal in individuals with the binocular dysfunction in a state of convergence deficiency and temporarily disrupting the somatosensory-proprioceptive loop signal related to postural control (Matheron & Kapoula, 2008). This mechanism can also be interpreted within broader theoretical frameworks explaining VR-induced discomfort. The findings align with both the sensory conflict theory and the postural instability theory, which provide complementary explanations for VR-related symptoms. Sensory conflict theory proposes that discrepancies between visually simulated motion and vestibular–proprioceptive inputs generate the perceptual discomfort underlying cybersickness, particularly disorientation. In contrast, postural instability theory emphasizes that sustained difficulty in maintaining stable posture within an unfamiliar virtual environment can precede and contribute to the onset of cybersickness. In this study, the combination of increased disorientation scores and greater postural sway among individuals with binocular dysfunction is consistent with both mechanisms, suggesting that challenges in sensory integration and reduced postural adaptability may jointly contribute to their heightened susceptibility. This interpretation is consistent with previous findings showing that excessive vergence demand negatively affects balance. Hwang (2023) stated that base-in prism correction in patients with exophoria was reported to be associated with improvements in body balance by eliminating the stress of continuous convergence to maintain binocular single vision, thereby restoring the neck muscle reflexes and eye movement signals, amongst others. These findings highlight that reducing sustained convergence effort in exophoric individuals can stabilize neuromuscular interactions involved in postural control. Although the mechanism differs from the vergence–accommodation imbalance induced by VR viewing, both situations suggest that individuals with limited fusional reserves may be more susceptible to postural instability when visual demands exceed their compensatory capacity. Moreover, our previous research (Jeong, 2024) underscored the importance of accurate refraction correction and to ensure that each user has the correct optical center distance of the VR device lens most comfortable for them. This adjustment may help reduce visual fatigue and support postural readjustment after viewing the VR video. Therefore, we propose inclusion of the following instructions in the comprehensive user manual for a VR device based on the findings of previous studies. (1) The cybersickness symptoms of a user who wears inappropriate corrective glasses may worsen after VR use. (2) Considering that the adjustment of the pupillary distance (PD) has the effect of neutralizing heterophoria, the user should adjust the headset to the most comfortable position to suit their eyes before viewing the VR video. Our suggestion is to design the distance between the lenses of the HMD device to be in the approximate range of 55 to 75 mm based on the distance between the pupils of the user’s eyes. (3) Individuals with frequent experiences of blurriness, intermittent double vision, or visual fatigue should be informed that cybersickness may become more severe after VR use. (4) Older adults should be instructed to use the VR device near a supportive object such as a safety bar because they need recovery time for postural adjustment immediately after viewing the VR video. These guidelines may contribute to improving safety and comfort by reflecting the characteristics of different VR users.

In recent years, despite the visual and tracking resolution of new HMD-VR technologies having greatly improved, users are still experiencing cybersickness and physical instability. The complex interaction of various factors seems to cause these effects, including the VR hardware (such as visual field, latency, and display resolution) (Gavgani et al., 2017; Shafer, Carbonara & Korpi, 2017; Garcia et al., 2017), the VR content (which encompasses graphic realism, reference frame, and optical flow) (Garcia et al., 2017; Chang, Kim & Yoo, 2020), and user parameters (including age, gender, susceptibility to sickness and previous VR experience) (Chang, Kim & Yoo, 2020). Furthermore, binocular dysfunction should be considered as an important human factor that increases cybersickness and postural instability. By performing additional studies, we aim to verify whether a VR-based training program for visual function would be effective for alleviating the cybersickness and postural instability in people with binocular dysfunction.

This study has several limitations that should be acknowledged. First, although pre–post differences following VR exposure were identified, the cross-sectional and correlational design does not allow causal relationships to be established among VR exposure, sensory conflict, cybersickness, and postural instability. Sensory conflict was not directly measured or manipulated; therefore, the proposed mechanistic pathway remains theoretical. Second, anthropometric factors such as height, weight, and stance width were not collected in this study, which limits the ability to account for their potential influence on postural stability. Third, binocular vision was evaluated at a single time point using clinical measures, and phoria, accommodation, and vergence were not reassessed immediately after VR exposure; therefore, changes in visual function due to VR exposure could not be examined directly. Furthermore, other subtypes of binocular dysfunction were not represented, which restricts the specificity and generalizability of the interpretation. Finally, the sample consisted of healthy young adults, which limits generalizability to older adults or clinical populations who may experience different levels of cybersickness or postural instability. Future research employing longitudinal or experimental manipulation of sensory conflict, along with more comprehensive visual and postural assessments, is needed to clarify causal pathways and broaden the applicability of the findings.

## Conclusions

This study aimed to identify the impact of binocular dysfunction on cybersickness and postural control in VR environments, providing essential reference data for VR technology development. Results showed that, compared to the normal group, the binocular dysfunction group exhibited increased exophoria at near, reduced amplitude of accommodative, and decreased positive fusional vergence at near. Additionally, symptoms of cybersickness and postural instability significantly increased following VR viewing. The binocular dysfunction group also demonstrated significantly higher oculomotor disorder and disorientation scores after VR exposure, with a positive correlation observed between greater cybersickness severity and increased the range anterior posterior of sway and the 95% confidence sway ellipse area.

These findings indicate that binocular dysfunction is associated with greater visual discomfort and postural instability in VR environments. Therefore, this study underscores the need for optical technologies and design adaptations in VR devices to address binocular dysfunction, which could enhance user comfort and safety in VR. Future research should consider various user characteristics, such as age and prior VR experience, to further investigate the effects of binocular dysfunction on cybersickness and postural control in VR environments.

##  Supplemental Information

10.7717/peerj.21169/supp-1 Supplemental Information 1 Raw data

## Additional Information and Declarations

### Data Availability

The following information was supplied regarding data availability: The raw data is available in the Supplemental File.
